# Digital Laser-Sintered Expander in Adolescent Patient with Hyperdontia and Molar Impaction

**DOI:** 10.1155/2023/8824900

**Published:** 2023-11-13

**Authors:** Greta Roussanova Yordanova-Kostova, Emanuel Emiliyanov, Nikolay Yanev

**Affiliations:** ^1^Department of Orthodontics, Faculty of Dental Medicine, Medical University of Sofia, Sofia, Bulgaria; ^2^University Hospital Medica Ruse, Ruse, Bulgaria

## Abstract

Supernumerary teeth can have normal or abnormal morphologic structure and characteristics, and their impacted form is diagnosed usually during X-ray examinations. In this case report, the presented patient is a 16-year-old female with anterior and right posterior open bite and bilateral posterior crossbite, upper right supernumerary paramolar, and impacted second and third molars. The paramolar development was the reason for the asymmetric growth of the alveolar bone in the upper jaw. The development of the bone is connected with the development of the teeth, and one additional tooth leads to extensive development in the maxilla. There is a risk of gingival recession occurrence when leveling the lower incisors due to the thin gingival biotype. A combined surgical-orthodontic treatment was done according to the following plan: extraction of supernumerary paramolar, germectomy of the upper right third molar (18) and at the same time periodontal graft in the lower anterior segment. A digitally three-dimensional (3D) printed appliance for rapid maxillary expansion was used for the transverse insufficiency of the upper jaw. The upper dental arch expander was designed with distal extension in the area of the upper right second molar (17). The extension was used as an anchorage during the orthodontic traction of the second molar. The treatment continued with a fixed orthodontic appliance—braces in the upper and lower jaw. With the extraction of the impacted and supernumerary teeth in the right maxillary segment, the eruption of 17 was stimulated and a change in the height of the alveolar bone was achieved. This favored the vertical changes and normalization of the occlusion. The maxillary expansion was also a significant factor in normalizing the occlusion. Observations on paramolar behavior showed that more often they develop in the bone and do not erupt. Each clinical case is highly individual, and patients seek orthodontic treatment at different stages of dentition development and corresponding development of the supernumerary teeth.

## 1. Introduction

Supernumerary teeth can have normal or abnormal morphologic structure and characteristics. Diagnosis of the erupted forms is relatively easy compared to the retention form. The impacted form is diagnosed usually during X-ray examinations. Supernumerary teeth are more frequent in the permanent dentition, and their frequency varies from 0.04% to 3.70% [1–3]. Sexual dimorphism is present in this biological phenomenon, more often affecting the male gender [4–7]. They are most commonly found in the maxilla. The area of the premaxilla is most affected by mesiodens—the typical representative of the supernumerary teeth. In the posterior segments, the supernumerary teeth can be parapremolar, paramolar, and distomolar. A paramolar is an additional molar that is usually small and rudimentary. It is located in buccal or palatal next to a molar and most often occurs in the interproximal spaces of the upper second and third molars [8–11]. Distomolars are defined as supernumerary teeth that develop distally from the third molar [12, 13].

Hyperdontia often aggravates the accompanying orthodontic deformity causing problems such as tooth retention, severe crowding, deviations in occlusal relationships, and delayed dentition development [14, 15]. Modern diagnostic such as cone beam computed tomography (CBCT) is characterized with accuracy and precision in establishing the localization and the stage of development of supernumerary teeth [16]. Some of the supernumerary teeth have late mineralization which makes it difficult to visualize them radiologically (they are round translucent blisters surrounded and limited by a poorly mineralized bone crypt of the tooth germ).

The impacted supernumerary teeth often cause disturbance in the eruption of the adjacent permanent teeth. In the literature, there are controversies regarding the optimal time for surgical removal of impacted supernumerary teeth [17]. When the hyperdontia is diagnosed in the mixed dentition, the surgical intervention increases the risk of damaging the permanent tooth germs, and as a result, their impaired eruption is observed [18]. However, in some patients, the extraction of the supernumerary teeth in the mixed dentition can stimulate the permanent tooth eruption. This is beneficial for their position in the dental arch and can reduce the need of future orthodontic treatment [9, 19]. Complications associated with surgical removal of impacted supernumerary teeth include luxation or fracture of adjacent teeth, root fracture, injuries to regional nerves (neuropathy), oroantral communications, and injuries (defects) of osseous structures.

## 2. Case Presentation

This study was approved by the Research Ethics Committee of Medical University, Sofia, logged under protocol number 04/12.05.2022.

The patient has signed standard informed consent and gave permission to use all of her data for the purpose of science research.

**Figure 1:**
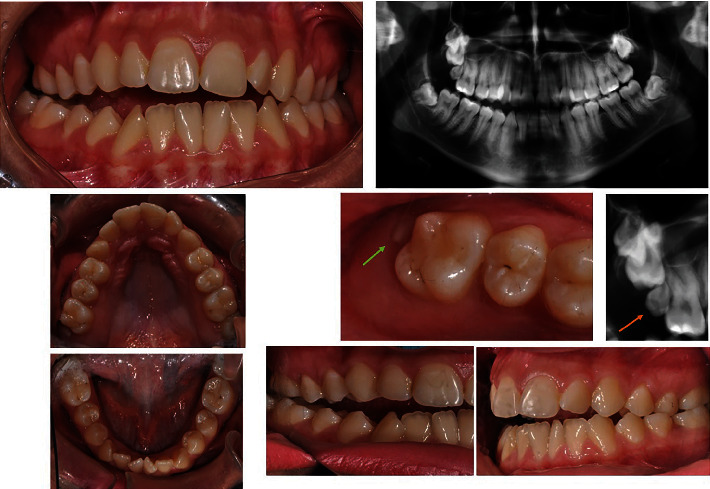
Intraoral photos and X-ray of the initial clinical condition: front side, X-ray, upper and lower jaw, right side, and left side. The pointer indicates the position of the supernumerary tooth.

The presented patient is a 16-year-old female with complaints about malocclusion which affects her speech. She was aimed for the orthodontic treatment by a speech therapist. In the first clinical examination, she was diagnosed with anterior and right posterior open bite and bilateral posterior crossbite (Figure 1). From the panoramic X-ray, upper right paramolar was detected. It was located between the first and second upper right molars (16 and 17). The supernumerary molar caused the impaction of the second upper right molar (17). The paramolar development was the reason for the asymmetric growth of the alveolar bone in the upper jaw. The development of the bone is connected with the development of the teeth, and one additional tooth leads to extensive development in the maxilla.

In the anamnesis, there was no evidence of family history. The general status of the patient indicated very good physical, skeletal, and intellectual development.

### 2.1. Orthodontic Diagnosis and Treatment Plan

After biometric and radiographic analysis of the patient, the composed treatment plan included extraction of the supernumerary tooth and the upper right third molar (18) in order to free the eruption path of 17. After that, a four-premolar extraction treatment was planned. The four-premolar extraction treatment is based on the need to close the open bite and the risk of gingival recession occurrence when leveling the lower incisors due to the thin gingival biotype. The patient was diagnosed with skeletal class I, dental crossbite in the distal segment, and with dental and skeletal open bite. The upper frontal teeth were protruded, and the lower frontal teeth were retruded.

The patient and her parents did not accept four-premolar extraction treatment. After discussing the likely benefits and risks of leveling the teeth in both dental arches and the treatment options for the open bite, a new treatment plan was developed. It included the following surgical and orthodontic stages:

1. Extraction of supernumerary paramolar
2. Germectomy of 18 (due to blocking the movement of the second molar, 17)
3. Periodontal graft in the lower anterior segment of the attached gingiva. The graft will be taken from the palate during the extraction of tooth 18
4. Rapid palatal expansion
5. Leveling of the teeth in both tooth arches
6. Closing the bite with intermaxillary elastics and correction of the occlusal plane (extrusion of the frontal teeth and intrusion of the distal teeth)

The graft surgery will stabilize the lower incisors and make possible the use of rotational movements. The extraction of 18 will be combined with surgical exposure of the impacted 17.

In order for the orthodontic traction of 17 to be possible, a palatal support in the molar area is needed. At the same time, the upper dental arch needs to be expanded, which will improve the occlusal relationships transversely and vertically. Therefore, it is appropriate to use a rapid maxillary expansion appliance with an added traction support element. Treatment will be completed with fixed appliance—braces and vertical intermaxillary elastics to normalize occlusal relationships. This was accepted from the patient and her parents and the fulfilled treatment plan. The alternative treatment plan for the patient was extraction of both teeth 17 and 18 and orthognathic surgery. This plan was not accepted from the patient and the parents.

The limitation is the lack of growth in this patient. The growth pick passed a few years before treatment started.

### 2.2. Treatment Course and Management

**Figure 2:**
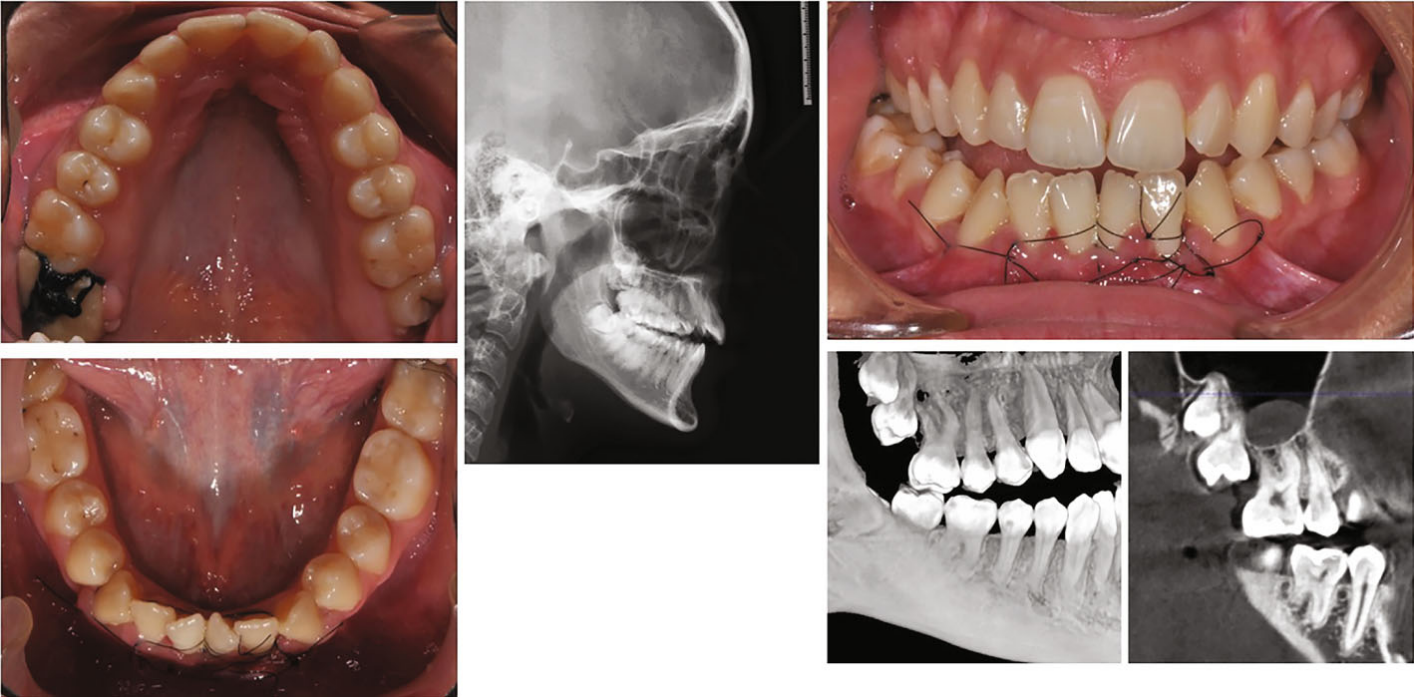
Extraction of supernumerary paramolar and then germectomy of the upper right third molar and periodontal graft in the lower anterior segment.

The extraction of the supernumerary paramolar was done taking all of the necessary precautions not to traumatize the adjacent teeth. After that, a supervisory CBCT examination was performed, and extraction of 18 was done, combined with the gingival graft surgery in the lower anterior segment (Figure 2). In the area of the donor mucosa, a temporary dressing was placed in order to preserve the exposure of the second molar until the digitally designed expander was made.

**Figure 3:**
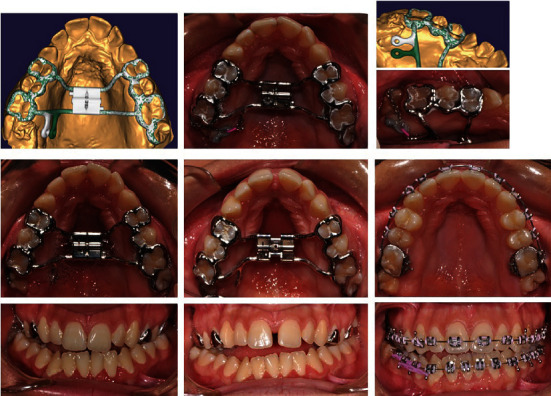
Follow-up of treatment stages: orthodontic traction of an impacted upper right second molar, rapid maxillary expansion, leveling of both dental arches, and normalization of occlusal relationships.

The upper dental arch expander was designed with distal extension in the area of 17. The extension was used as an anchorage during the orthodontic traction of the second molar. The design of the appliance was made with exocad software and was printed with CAM technology from Co-Cr alloy by laser sintering of the metal. The appliance was bonded to the distal teeth in the maxilla (Figure 3). The orthodontic traction and positioning of the impacted 17 into the dental arch was performed first. The biomechanics of the traction are possible because of the good anchorage zone (teeth 16, 15, 14, 24, 25, 26, and 27 were incorporated to one anchorage union). The customized appliance allowed the traction to be in palatal direction as possible. The force is maximally close to the center of resistance of tooth 17. This prevented any unwanted rotation during traction. After tooth 17 was positioned and stabilized in the arch, the expander activation started. The expansion screw was activated once a day for 24 days straight. One activation corresponds to quarter of a turn of the screw. It was done by the parents at the same time each day. Optimal transverse contacts were achieved between the posterior upper and lower teeth. After the expansion, there was a 2-month retention phase, and then, the expander was removed. The treatment continued with a fixed orthodontic appliance—braces in the upper and lower jaw. The patient strictly followed the instructions given to him and correctly placed the intermaxillary elastics. Later, a germectomy of the lower right third molar was performed.

**Figure 4:**
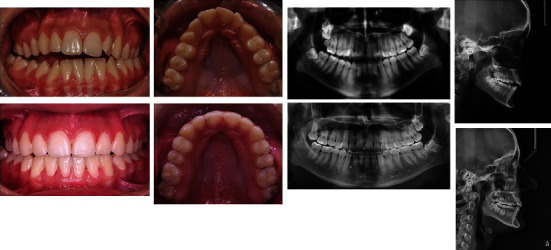
Intraoral photos and X-rays of the initial and finishing condition.

With the extraction of the impacted and supernumerary teeth in the right maxillary segment, the eruption of 17 was stimulated and a change in the height of the alveolar bone was achieved (Figure 4). This favored the vertical changes and normalization of the occlusion. The maxillary expansion was also a significant factor in normalizing the occlusion.

**Figure 5:**
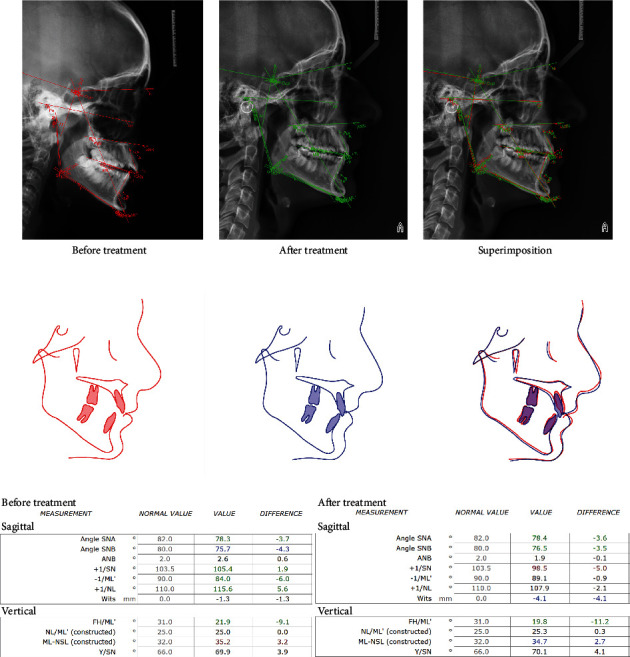
Change in dental-alveolar and skeletal parameters.

As a result of the orthodontic treatment, all dental-alveolar and skeletal parameters were improved (Figure 5). Normalization of occlusal relationships in all planes was achieved. The lower front teeth were well leveled without recessions and soft tissue loss.

### 2.3. Follow-Up

**Figure 6:**
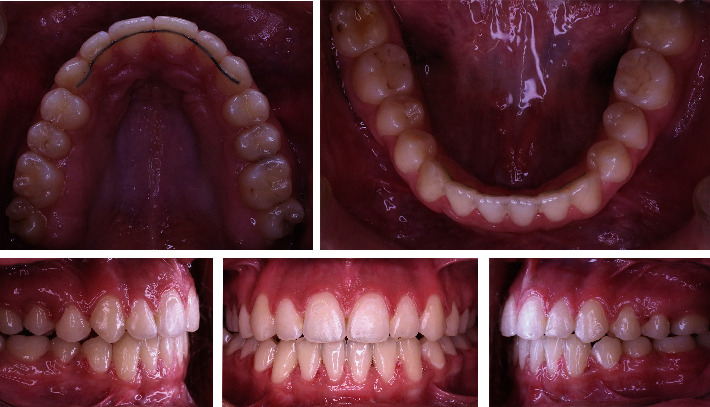
Intraoral follow-up photos.

The orthodontic treatment was successful, without any complications. After finishing the treatment, there were no recessions and there is no open bite. The occlusion stayed in its normal relations. The patient was followed up for two years during the retention phase (Figure 6). In this phase, fixed retention appliances (fixed retainers) were bonded in the maxilla and mandible.

## 3. Discussion

The most frequently reported clinical cases in the available academic literature associate hyperdontia with a hereditary predisposition, whereas in the presented case, no such association was established with any of the patient's relatives [20, 21].

The presented clinical case does not correspond with the typical localization of the supernumerary teeth (usually in the premaxilla) and the more frequent occurrence in males. The presence of a paramolar between 16 and 17 was the reason for the impaction of 17 and 18. This led to the need for extraction of the third molar and orthodontic traction of the second molar. These extractions were performed consecutively, because there is a high risk for the adjacent teeth when extracting a supernumerary tooth.

The time of the surgical intervention is important and is related to the possibility of deleterious effects on the dental-alveolar structures [20]. Early diagnosis of supernumerary impacted teeth and their localization and connection with the surrounding bone structures is the most important factor for determining the outcome of the surgical intervention [20].

Other authors report the danger of fusion of the supernumerary teeth with adjacent tooth structures at the crown or root level, which can make the extraction more difficult [21, 22]. This type of tooth morphology is a real cause of occlusal relationship disturbances, crowding in the posterior segments of the dental arch, predisposition to caries, and periodontal diseases [23].

Another clinical approach is observation of the supernumerary teeth without extraction. This approach is suitable in case that the adjacent teeth erupt without any associated pathology, functional, and aesthetic disturbances [22]. In the presented clinical case, this approach was not appropriate because tooth 17 was impacted and there were disturbances in the alveolar bone development and in the occlusal relationships.

Observations on paramolar behavior showed that more often they develop in the bone and do not erupt. They are discovered as an incidental radiographic finding because clinical signs are often absent [24, 25]. Furthermore, supernumerary teeth in the premolar region often develop late compared to the normal teeth and sometimes even after the orthodontic treatment has been finished [25]. The treatment is most often conservative and includes extraction of the supernumerary tooth [20]. The main motive for supernumerary tooth extraction is the lack of space in the dental arch to level the permanent teeth. This was one of the reasons for the extraction in the presented clinical case. Therefore, the extraction of the paramolar solved the crowding problem in the posterior segment and the occlusal disturbances. The critical moment for the maxillofacial surgeon was the root development stage of the adjacent teeth (first and second molars), which was radiographically confirmed to be complete. In the presented clinical case, the supernumerary paramolar was discovered late—at the age of 16. The authors who researched hyperdontia in the premaxilla suggest that orthodontic treatment can be postponed for a few months after the extraction of the supernumerary tooth as potential self-correction can be expected [26]. Examinations by the family dentist should have established the retained eruption of the upper right second molar, and radiological diagnostics should have been carried out searching for a mechanical reason for this retention. Therefore, the early diagnosis is important in order not to aggravate the developing orthodontic deformity as a result of the hyperdontia [20].

Each clinical case is highly individual, and patients seek orthodontic treatment at different stages of dentition development and corresponding development of the supernumerary teeth. Development of digital orthodontic appliances designed and manufactured with CAD/CAM technology expanded the possible clinical solutions. Printed expanders allow planning of the direction of the acting force. This makes the result more predictable [27].

## 4. Conclusion

The supernumerary teeth are rare disorder, but if they are found on time, the development of orthodontic deformation can be prevented. We recommend preventive examinations by the family dentists. They should be done with a mandatory X-ray examination at least in the two main stages of dentition development—early mixed dentition and the initial period of the permanent dentition. This early screening would detect a change in the number of the permanent teeth in the anterior and posterior segments, which would prevent aggravation of the orthodontic deformity. If the family dentist finds a supernumerary tooth, he or she should recommend immediate orthodontic consultation. This disorder can lead to changes in maxillofacial growth and development which may cause asymmetry in craniofacial development and severe orthodontic and surgery problems. The individual orthodontic approach is necessary in each case. Customization of the appliances makes the treatment strategy more flexible, creates an opportunity to include new anchorage mechanisms, and creates predictable results. The printed appliances and support grids are well received by patients. They are comfortable due to their exceptional accuracy to the teeth and the surrounding structures (palate, alveolar ridge, or mucosa). Good appliance planning makes the orthodontist's job easy and reduces the risk of human error.

## Data Availability

The data are completely presented as the case report.

## References

[1] Palikaraki G., Vardas E., Mitsea A. (2019). Two rare cases of non-syndromic paramolars with family occurrence and a review of literature. *Dentistry Journal*.

[2] Brinkmann J. C., Martínez-Rodríguez N., Martín-Ares M. (2020). Epidemiological features and clinical repercussions of supernumerary teeth in a multicenter study: a review of 518 patients with hyperdontia in Spanish population. *European Journal of Dentistry*.

[3] Hajmohammadi E., Najirad S., Mikaeili H., Kamran A. (2021). Epidemiology of supernumerary teeth in 5000 radiography films: investigation of patients referring to the clinics of Ardabil in 2015-2020. *International Journal of Dentistry*.

[4] Srivatsan P., Aravindha Babu N. (2003). Mesiodens with an unusual morphology and multiple impacted supernumerary teeth in a non-syndromic patient. *Indian Journal of Dental Research*.

[5] Polder B. J., Van't Hof M. A., Van der Linden F. P., Kuijpers-Jagtman A. M. (2004). A meta-analysis of the prevalence of dental agenesis of permanent teeth. *Community Dentistry and Oral Epidemiology*.

[6] Demiriz L., Durmuşlar M. C., Mısır A. F. (2015). Prevalence and characteristics of supernumerary teeth: a survey on 7348 people. *Journal of International Society of Preventive & Community Dentistry*.

[7] Celikoglu M., Kamak H., Oktay H. (2009). Prevalence and characteristics of supernumerary teeth in a non-syndrome Turkish population: associated pathologies and proposed treatment. *Medicina Oral Patologia Oral y Cirugia Bucal*.

[8] Nayak G., Shetty S., Singh I., Pitalia D. (2012). Paramolar - a supernumerary molar: a case report and an overview. *Dental Research Journal*.

[9] Tworkowski K., Gąsowska E., Baryła D., Gabiec K. (2020). Supernumerary teeth – literature review. *Journal of Pre-Clinical and Clinical Research*.

[10] Sulabha A. N., Sameer C. (2015). Unusual bilateral paramolars associated with clinical complications. *Case Reports in Dentistry*.

[11] Gutierrez-Marín N. (2021). Non-erupted maxillary bilateral paramolars and their surgical approach: unusual case report. *Revista de Odontopediatría Latinoamericana*.

[12] Kaya E., Güngör K., Demirel O., Özütürk Ö. (2015). Prevalence and characteristics of non-syndromic distomolars: a retrospective study. *Journal of Investigative and Clinical Dentistry*.

[13] Di Donna E., Keller L. M., Neri A., Perez A., Lombardi T. (2022). Maxillary distomolar associated with dentigerous cyst: an unusual entity. *Oral*.

[14] Cholakova R. (2020). Clinical and epidemiological study of supernumerary teeth in patients from Plovdiv region. *Acta Medica Bulgarica*.

[15] Mitchell L., Bennett T. G. (1992). Supernumerary teeth causing delayed eruption—a retrospective study. *British Journal of Orthodontics.*.

[16] Ata-Ali F., Ata-Ali J., Peñarrocha-Oltra D., Peñarrocha-Diago M. (2014). Prevalence, etiology, diagnosis, treatment and complications of supernumerary teeth. *Journal of Clinical and Experimental Dentistry*.

[17] Omer R. S., Anthonappa R. P., King N. M. (2010). Determination of the optimum time for surgical removal of unerupted anterior supernumerary teeth. *Pediatric Dentistry*.

[18] Russell K. A., Folwarczna M. A. (2003). Mesiodens — diagnosis and management of a common supernumerary tooth. *Journal of the Canadian Dental Association*.

[19] Tworkowski K., Gąsowska E., Baryła D., Gabiec K. (2020). Supernumerary teeth – literature review. *Journal of Pre-Clinical and Clinical Research*.

[20] Gupta S., Marwah N. (2012). Impacted supernumerary teeth-early or delayed intervention: decision making dilemma?. *International journal of clinical pediatric dentistry*.

[21] Moradinejad M., Hashemi Ashtiani A., Rakhshan V. (2022). Multiple nonsyndromic unerupted supernumerary teeth: a report of a rare case. *Case Reports in Dentistry*.

[22] Parolia A., Kundabala M., Dahal M., Mohan M., Thomas M. S. (2011). Management of supernumerary teeth. *Journal of Conservative Dentistry*.

[23] Ren S., Miao L., Yu Y., Zhang X., Zhu M., Sun M. (2016). Fusion between maxillary second molar and a supernumerary tooth: a case report. *International Journal of Clinical and Experimental Medicine*.

[24] Solares R., Romero M. I. (2004). Supernumerary premolars: a literature review. *Pediatric Dentistry*.

[25] Khalaf K., Al Shehadat S., Murray C. A. (2018). A review of supernumerary teeth in the premolar region. *International journal of dentistry*.

[26] Cholakova R., Georgiev K. (2023). Effect of maxillary anterior supernumerary tooth extraction on the underlying malocclusion. *Journal of Orthodontics*.

[27] Petrunov V. (2020). Rapid palatal expander made using direct metal laser sintering technology. *IV Congress of BAOS–eBook*.

